# Independent and interactive associations of inflammation, vascular homeostasis markers, and childhood trauma with suicide attempt history

**DOI:** 10.1192/j.eurpsy.2025.10029

**Published:** 2025-05-27

**Authors:** Ainoa García-Fernández, Aiste Lengvenyte, Elia Gourguechon-Buot, Emilie Olié, Fabrice Cognasse, Pilar A. Sáiz, Philippe Courtet

**Affiliations:** 1 Department of Psychiatry, University of Oviedo, Oviedo, Spain; 2 Instituto de Investigación Sanitaria del Principado de Asturias (ISPA), Oviedo, Spain; 3 Instituto Universitario de Neurociencias del Principado de Asturias (INEUROPA), Oviedo, Spain; 4 Centro de Investigación Biomédica en Red de Salud Mental (CIBERSAM), Madrid, Spain; 5 Department of Emergency Psychiatry and Acute Care, Lapeyronie Hospital, CHU Montpellier, Montpellier, France; 6 IGF, University of Montpellier, CNRS, INSERM, Montpellier, France; 7 Servicio de Salud del Principado de Asturias (SESPA), Oviedo, Spain.

**Keywords:** biomarker, childhood trauma, depression, inflammation, suicide attempt

## Abstract

**Background:**

The neurobiological basis of suicidal behaviour remains poorly understood. However, emerging evidence suggests that inflammation and vascular homeostasis factors may play a role in its pathophysiology. Childhood trauma, through immune system dysfunction and increased risk of suicidal behaviours, might influence these associations. This study examined the relationships between immune-inflammatory and vascular homeostasis-related markers and their interaction with childhood trauma in relation to a history of suicide attempts in individuals with depression.

**Methods:**

A total of 328 patients with major depression were recruited: 166 with a history of suicide attempts and 162 without. Using multivariate binary logistic regression models adjusted for cofounders, we examined the associations between childhood trauma, levels of platelet-related immune markers (serotonin, MCP-1, TSP-1, TSP-2, PDGF-AB, PDGF-BB), and suicide attempt history. Independent associations between PDGF-BB, childhood trauma, and suicide attempts were further assessed using interaction models. Stratified sensitivity analyses based on childhood trauma history were also conducted.

**Results:**

Childhood trauma consistently emerged as associated with suicide attempts across all models. Among the measured biomarkers, higher TSP-2 levels were associated with a suicide attempt history, independent of childhood trauma. Meanwhile, while PDGF-BB alone was not directly linked to suicide attempt history, the interaction analysis revealed that individuals with lower PDGF-BB levels and more severe childhood trauma were more likely to have attempted suicide.

**Conclusions:**

TSP-2 and PDGF-BB are potential biomarkers linked to suicide attempts, with distinct roles in the interplay between biological processes and early-life adversity. These insights can inform the biomarker-informed development of tailored prevention and treatment strategies.

## Introduction

Suicide attempts (SA) in patients with mood disorders are among the strongest predictors of future attempts and completed suicides [1], with devastating consequences for individuals, families, and society. Approximately 60% of suicides are linked to depression [2]. This underscores the urgent need to identify biological, clinical, and environmental factors associated with suicidal behaviours in people with depression, and to understand their interactions.

One of the leading theoretical models of suicide, the stress-diathesis model, proposes that suicidal behaviour results from an interaction between distal risk factors, such as genetic predisposition and early life adversity, and acute stressors. Within this framework, biological correlates of distal risk factors may serve as potential biomarkers for individual predisposition to suicidal behaviours [3].

In this context, childhood adversity has emerged as a key distal risk factor for suicidal behaviours, increasing the likelihood of both psychiatric conditions and SA in people with these conditions [4, 5]. Individuals with a history of childhood trauma are nearly three times more likely to report SA [6], with emotional and sexual abuse identified as particularly strong predictors [7]. Notably, adverse experiences in early life are not only linked to suicidal behaviour but also immune dysregulation, which has been proposed as the potential underlying mechanism linking childhood trauma to increased suicide risk [8]. Studies have also reported an additive effect of genetic variations in genes implicated in oxidative stress and vascular homeostasis, together with childhood trauma, on SA [9]. Emerging evidence suggests a potential interaction between neuroinflammation and childhood trauma in increasing the risk of suicidal behaviours [10, 11].

Extensive research has demonstrated that immune dysfunction is observed in a substantial portion of individuals with mood disorders [12]. Although several predictive biomarkers for depression severity and suicidal behaviours have been proposed, findings, particularly those concerning causality, remain inconsistent. Early studies reported that genetic variations in interleukin-1 (IL-1) or tumour necrosis factor-alpha (TNF-alpha) did not influence the susceptibility to suicidal behaviours [13]. However, increased IL-6 and C-reactive protein (CRP) levels have been consistently associated with suicidal behaviour in people with depression and anxiety, independently of depression severity [14, 15]. Changes in other inflammatory markers, such as decreased IL-2, have also been observed in depressed suicide attempters compared to depressed individuals without a history of SA [16–18]. Beyond traditional inflammatory markers, elevated levels of nitro-oxidative stress markers and certain growth factors have also been associated with suicidal ideation and behaviour [19].

Despite these advances, the biological mechanisms underlying suicidal behaviour remain incompletely understood, and novel pathways continue to emerge. Recent research has identified vascular homeostasis-related markers thrombospondins and platelet-derived growth factors as potential diagnostic and predictive biomarkers for suicidal events [20]. However, to the best of our knowledge, no previous studies have examined the interaction between these novel biomarkers and childhood adversity in relation to suicidal behaviours. To bridge these gaps, the present study aimed (i) to investigate the association between soluble platelet-related biomarkers and lifetime suicide attempts in individuals with depression and (ii) to determine whether these biological markers and childhood adversity are jointly associated with increased likelihood of suicidal behaviours. We hypothesized that higher or lower levels of certain soluble platelet-related biomarkers would be associated with a history of suicide attempts in individuals with depression and that these associations would be stronger in individuals reporting childhood trauma. Furthermore, we anticipated that combining biomarker data with childhood adversity would help better identify the individuals with a history of suicide attempts compared to examining each factor alone. Understanding these interactions may enhance our understanding of the biological mechanisms underlying suicidal behaviour and contribute to the development of predictive tools and targeted interventions.

## Material and methods

### Procedure and participants

This is a secondary analysis of a database collected in the Psychiatric Emergency Department and Acute Care of Montpellier University Hospital, France, from three studies (NCT04137458, NCT02824081, and NCT02710279) that explored biological correlates of suicidal behaviours that were conducted between January 2016 and July 2022. All three studies were carried out according to the ethical principles of the Declaration of Helsinki and the Good Clinical Practice guidelines and were approved by the local Ethics Committee (CPP Sud Mediterranée IV, CHU Montpellier, France). The studies also used the same key set of inclusion and exclusion criteria and allowed for secondary data analysis, which was stated in the consent, signed by study participants.

Patients were recruited at the Psychiatric Emergency Department and Acute Care of Montpellier University Hospital (France), which specializes in mood disorders and suicidal thoughts and behaviours. A total of 328 patients diagnosed with a major depressive episode were included and classified into the following groups: (1) individuals with a major depressive episode and a lifetime history of SA (*N* = 166); (2) individuals with a major depressive episode without a history of SA (*N* = 162 individuals without SA). Inclusion criteria were: age between 18 and 65 years, a current diagnosis of major depressive episode according to the Diagnostic and Statistical Manual 5 (DSM-5) criteria, having completed the Childhood Trauma Questionnaire-Short Form (CTQ-SF), and the ability to understand the research protocol and provide informed consent. Exclusion criteria were: acute inflammatory conditions (either symptomatic or high sensitivity C-reactive protein (hsCRP) >50 ng/ml), current anti-inflammatory or immunomodulatory treatment, pregnancy or breastfeeding, current psychotic symptoms, difficulty understanding the study’s objectives, lack of social security, participation in another study, refusal to participate, or deprivation of liberty due to judicial or administrative decisions.

### Assessments

All patients were assessed by trained clinicians. The assessment included the administration of an ad hoc questionnaire designed to collect sociodemographic and clinical information, treatment, and history of SA. SA history was defined as a self-initiated, potentially injurious behaviour with a nonfatal outcome, accompanied by evidence (explicit or implicit) of intent to die. Psychiatric diagnoses were assessed by an experienced psychiatrist with a Structured Clinical Diagnostic Interview for DSM-5. The CTQ-SF was used to assess childhood trauma history. It is a self-report retrospective scale designed to assess trauma during childhood and adolescence [21]. CTQ-SF contains 28 items, of which 25 are grouped into five subscales: emotional abuse, physical abuse, sexual abuse, emotional neglect, and physical neglect. Each item is rated on a 5-point Likert-type scale from 1 (never) to 5 (almost always), with higher scores indicating greater childhood trauma severity. Total scores and subscores for childhood emotional abuse and neglect were used. The Beck Depression Inventory (BDI) was used to assess current depression severity [22]. BDI is a self-report questionnaire that contains 21 items rated on a 4-point Likert-type scale from 0 to 3. The total score ranges from 0 to 63, and higher scores reflect higher symptom severity. Finally, the Psychological-Physical-Pain Visual Analogue Scale (PPP-VAS) is a self-administered questionnaire for measuring psychological and physical pain. The VAS is a straight horizontal line labelled “no pain” on the left (score = 0) and “worst pain” on the right (score = 10) [23].

### Covariates

The initial analysis included the following covariates: sex, age, and body mass index (BMI). The following covariates were also considered in subsequent models: current psychiatric diagnoses, including bipolar disorder, anxiety disorders, eating disorders, and substance use disorders or alcohol use disorders; current tobacco use, and psychiatric pharmacological treatment (antidepressants, antipsychotics, anxiolytics, antiepileptics, and lithium).

### Blood analysis

Blood samples were taken within 24 h after the initial evaluation. Fasting venous blood samples were collected after an overnight fast between 8:00 AM and 10:00 AM. Blood samples were collected in endotoxin-free 3.8% sodium citrate tubes (Vacutainer®, Becton-Dickinson) and handled by the hospital technical staff. The high-sensitivity C-reactive protein (hsCRP) was included as a classical inflammatory marker most robustly associated with suicidal behaviour in depression [14]. It was analysed on a Roche C800 platform on the same day. In addition, a broader involvement of platelet biological responses and vascular homeostasis has been recently suggested to be implicated in suicidal behaviours [20]. We therefore examined the following platelet-related markers: platelet-derived growth factor (PDGF)-AB, PDGF-BB, thrombospondin (TSP)-1, TSP-2, monocyte chemoattractant protein-1 (MCP-1) and serotonin. For the protein analyses, plasma was separated by centrifugation at 3000×*g* for 20 min at room temperature and stored at −80°C for subsequent batched analysis using ELISA assays. All parameters were assayed in duplicate wells. We used Merch Millipore (Luminex), Bio-Techne SA (Chatillon sur Seiche, France), and Tecan France (Lyon, France) (ELISA) platforms. Inter-plate variability was low for all analytes (coefficient of variation (CV) < 2). All biological variables were log-transformed and standardized within each included study to avoid study effects. The undetectable values were imputed separately for each study using a censored log-normal imputation.

### Data analysis

Data were first assessed for normality, and variables with skewed distribution were log-transformed to achieve acceptable skewness and kurtosis values. Descriptive statistics are reported as frequencies and percentages for categorical variables and means and standard deviations (SD) for continuous variables. Initial bivariate analyses were conducted using the Chi-square test for categorical variables and the Wilcoxon rank-sum test for continuous variables. Pairwise Spearman rank correlations were used to examine the relationships between continuous variables.

To assess the associations between biological markers, childhood trauma, and SA history, we employed multivariate binary logistic regression models. The baseline model (Model 1) consisted of assessing the associations between each of the immune markers, childhood trauma severity (predictors), and SA history (outcome), adjusted for age, sex, and BMI. In Model 2 (interaction model), we explored interactions between each immune marker and childhood trauma severity with respect to the SA history, using the same covariates and including the interaction term. Two additional models were constructed to test the robustness of the observed associations in Models 1 and 2. In addition to baseline adjustment, Model 3 further incorporated adjustments for depression severity and psychiatric diagnoses, while Model 4 additionally controlled for current tobacco use and major psychopharmacological treatment classes. In addition, we applied binomial logistic regression models to assess the associations of CTQ-SF scores and PDGF-BB levels with SA history, examining each factor separately, in combination, and through an interaction model, adjusting for sex, age, BMI, and tobacco use.

As a sensitivity analysis, we performed a stratified analysis to assess the association between immune markers and SA history in individuals with a history of at least one type of childhood trauma versus those without such a history. The same models were used in this stratified analysis, except the interaction term was omitted.

All statistical analyses were performed using R statistical computing software, version 4.3.0 (R Foundation for Statistical Computing, Vienna, Austria). Statistical significance was set at a two-tailed *p* < 0.05. To account for multiple comparisons, the Benjamini–Hochberg False Discovery Rate (FDR) correction (*q* < 0.05) [24] was applied to family-wise regression analyses.

## Results

### Sample characteristics and group comparisons

A total of 328 patients aged 18–65 years were included, with a mean age of 38 years (SD = 12.5). The sample comprised 71.64% females (*N* = 235). Table 1 presents the demographic and clinical characteristics of the sample, comparing patients with a history of SA (*n* = 166) to those without (*N* = 162). Suicide attempters had a significantly higher proportion of females (*p* = 0.048) and single individuals (*p* < 0.001) compared to non-attempters. They also had fewer years of education (*p* = 0.010) and were less likely to have a professional activity (*p* = 0.044). Individuals with a SA history were also more likely to have a current mood disorder (*p* = 0.010), past-year alcohol use disorder (*p* = 0.022), lifetime eating disorder (*p* = 0.025), and a family history of suicide or SA (*p* = 0.009). Regarding psychiatric treatment, patients with a SA history were prescribed more anxiolytics (*p* < 0.028) and antipsychotics (*p* = 0.007). Additionally, patients with a history of SA reported more current tobacco use (*p* = 0.011) and had higher depression severity (*p* < 0.001) and scores across several dimensions of childhood trauma, including physical neglect (*p* = 0.005), emotional abuse (*p* < 0.001) and emotional neglect (*p* < 0.001). They also reported higher levels of psychological pain (*p* < 0.001), physical pain (*p* = 0.011), and suicidal ideation (*p* < 0.001). Plasma biomarker analysis revealed that participants with SA history had higher plasma levels of MCP-1 (*p* = 0.047) and TSP-2 (*p* = 0.050), and lower plasma levels of serotonin (*p* = 0.007).

**Table 1.** Sociodemographic, clinical, and biological features of the sample between patients with or without suicide attempt history

	Non-SA group*N* = 162	SA group*N* = 166	*p*-Value^a^
Gender, *n* (%)			**0.048**
Female	108 (67 %)	127 (77 %)	
Male	54 (33 %)	39 (23 %)	
Age; median (IQR); mean (SD)	40 (17) 40 (12)	38 (23) 38 (13)	0.13
Single, *n* (%)			**<0.001**
No	89 (55 %)	51 (31 %)	
Yes	73 (45 %)	112 (69 %)	
Study level (years); median (IQR); mean (SD)	14.00 (4.00) 14.27 (2.34)	14.00 (3.00) 13.52 (2.55)	**0.010**
Professional activity, *n* (%)			**0.044**
No	85 (53 %)	104 (64 %)	
Yes	76 (47 %)	59 (36 %)	
Major depressive episode (current), *n* (%)			**0.010**
No	42 (26 %)	24 (14 %)	
Yes	120 (74 %)	142 (86 %)	
Major depressive episode (past), *n* (%)			0.6
No	14 (8.6 %)	12 (7.2 %)	
Yes	148 (91 %)	154 (93 %)	
Bipolar disorder (lifetime), *n* (%)			0.3
No	97 (60 %)	108 (65 %)	
Yes	65 (40 %)	58 (35 %)	
Alcohol dependence/abuse (current), *n* (%)			**0.022**
No	143 (88 %)	131 (79 %)	
Yes	19 (12 %)	35 (21 %)	
Substance dependence/abuse (current), *n* (%)			0.2
No	146 (90 %)	142 (86 %)	
Yes	16 (9.9 %)	24 (14 %)	
Eating disorder (lifetime), *n* (%)			**0.025**
No	142 (88 %)	130 (78 %)	
Yes	20 (12 %)	36 (22 %)	
Anxiety disorder, *n* (%)			0.6
No	48 (30 %)	45 (27 %)	
Yes	114 (70 %)	121 (73 %)	
Family history of suicide, *n* (%)			>0.9
No	131 (81 %)	135 (81 %)	
Yes	31 (19 %)	31 (19 %)	
Family history of suicide attempt, *n* (%)			**0.009**
No	134 (83 %)	117 (70 %)	
Yes	28 (17 %)	49 (30 %)	
Family history of suicide or suicide attempt, *n* (%)			0.058
No	111 (69 %)	97 (58 %)	
Yes	51 (31 %)	69 (42 %)	
Body mass index; median (IQR); mean (SD)	23.1 (6.8) 23.8 (4.9)	23.7 (6.5) 24.3 (5.4)	0.4
Metabolic syndrome, *n* (%)			0.2
*No*	145 (92 %)	134 (87 %)	
*Yes*	13 (8.2 %)	20 (13 %)	
Current tobacco usage, *n* (%)			**0.011**
*No*	96 (59 %)	75 (45 %)	
*Yes*	66 (41 %)	91 (55 %)	
Psycholeptics (anxiolytics/hypnotics/antipsychotics), *n* (%)			**0.007**
No	50 (31 %)	30 (18 %)	
Yes	112 (69 %)	136 (82 %)	
Anxiolytics, *n* (%)			**0.028**
No	77 (48 %)	59 (36 %)	
Yes	85 (52 %)	107 (64 %)	
Antidepressants, *n* (%)			0.2
No	65 (40 %)	56 (34 %)	
Yes	97 (60 %)	110 (66 %)	
Antiepileptics, *n* (%)			0.14
No	131 (81 %)	123 (74 %)	
Yes	31 (19 %)	43 (26 %)	
Antipsychotics, *n* (%)			0.3
No	86 (53 %)	79 (48 %)	
Yes	76 (47 %)	87 (52 %)	
Lithium, *n* (%)			0.8
No	146 (90 %)	148 (89 %)	
Yes	16 (9.9 %)	18 (11 %)	
Analgesics, *n* (%)			
No	162 (100 %)	166 (100 %)	
Antiinflammatories, *n* (%)			
No	162 (100 %)	166 (100 %)	
PPP-VAS emotional pain	8.00 (3.00) 6.99 (3.19)	9.00 (2.00) 8.40 (2.08)	**<0.001**
PPP-VAS physical pain	3.0 (7.0) 3.8 (3.4)	5.0 (5.3) 4.8 (3.3)	**0.011**
PPP-VAS suicidal thoughts	1.0 (8.0) 3.4 (4.0)	8.0 (4.0) 7.1 (3.5)	**<0.001**
BDI score; median (IQR); mean (SD)	14 (12) 14 (8)	18 (11) 18 (8)	**<0.001**
CTQ-SF total score; median (IQR); mean (SD)	42 (24) 46 (16)	52 (25) 54 (19)	**<0.001**
CTQ-SF physical abuse; median (IQR); mean (SD)	6.0 (3.0) 7.3 (3.5)	6.0 (5.0) 8.6 (5.3)	0.3
CTQ-SF physical neglect; median (IQR); mean (SD)	7.0 (4.0) 7.9 (3.1)	8.0 (5.0) 9.0 (3.6)	**0.005**
CTQ-SF emotional abuse; median (IQR); mean (SD)	9.0 (9.0) 11.0 (5.2)	14.0 (10.8) 13.6 (5.8)	**<0.001**
CTQ-SF emotional neglect; median (IQR); mean (SD)	13.0 (9.0) 12.9 (5.7)	16.0 (8.0) 15.0 (5.4)	**0.001**
CTQ-SF sexual abuse; median (IQR); mean (SD)	5.0 (2.0) 7.4 (4.8)	5.0 (4.0) 8.1 (5.2)	0.087
CTQ-SF physical abuse (moderate or severe)			**0.030**
No	124 (82 %)	105 (71 %)	
Yes	28 (18 %)	43 (29 %)	
CTQ-SF physical negligence (moderate or severe)			0.069
No	116 (75 %)	96 (66 %)	
Yes	38 (25 %)	50 (34 %)	
CTQ-SF emotional abuse (moderate or severe)			**0.009**
No	88 (60 %)	67 (45 %)	
Yes	59 (40 %)	83 (55 %)	
CTQ-SF emotional negligence (moderate or severe)			**0.031**
No	87 (59 %)	69 (46 %)	
Yes	61 (41 %)	80 (54 %)	
CTQ-SF sexual abuse (moderate or severe)			0.2
No	116 (75 %)	101 (69 %)	
Yes	38 (25 %)	46 (31 %)	
CTQ-SF at least one form of moderate abuse			**<0.001**
No	61 (41 %)	33 (22 %)	
Yes	88 (59 %)	115 (78 %)	
Serotonin; median (IQR); mean (SD)	0.00 (1.42) 0.08 (0.99)	−0.10 (1.39) −0.08 (1.00)	0.2
Monocyte chemoattractant protein–1 (MCP–1); median (IQR); mean (SD)	0.30 (1.63) −0.13 (1.04)	0.47 (1.36) 0.13 (0.94)	**0.047**
Platelet-derived growth factor (PDGF-AB); median (IQR); mean (SD)	0.12 (1.23) 0.00 (0.98)	0.21 (1.37) 0.00 (1.01)	0.8
Platelet-Derived Growth Factor (PDGF-BB); median (IQR); mean (SD)	0.34 (1.75) −0.02 (1.01)	0.42 (1.07) 0.03 (0.99)	0.8
Thrombospondin 1 (TSP–1); median (IQR); mean (SD)	0.14 (1.70) 0.04 (1.00)	0.05 (1.50) −0.03 (0.99)	0.5
Thrombospondin 2 (TSP–2); median (IQR); mean (SD)	−0.11 (1.52) −0.15 (1.02)	0.22 (1.19) 0.15 (0.96)	**0.010**
C-reactive protein (CRP); median (IQR); mean (SD)	−0.23 (1.47) −0.09 (0.98)	0.24 (1.71) 0.09 (1.01)	0.093

a Differences between the groups were tested with either a chi-squared test (dichotomous) or a Wilcoxon rank sum test (continuous).

Abbreviations: BDI, Beck Depression Inventory; CTQ-SF, Childhood Trauma Questionnaire-Short Form; PPP-VAS, Psychological-Physical-Pain Visual Analogue Scale.

Bolded values correspond to *p*-values <0.05, two-sided.

Partial Spearman regression between biological markers, childhood trauma, and depression severity, adjusted for age, sex, BMI, and current tobacco use, showed that lower plasma levels of serotonin (OR = −0.225, 95% CI: −0.331, −0.114, *p* < 0.001) and PDGF-BB (OR = −0.109, 95% CI: −0.217, 0, *p* = 0.005) were modestly, but significantly associated with higher depression severity scores (Supplementary Table S1). Conversely, childhood trauma scores were not correlated with any of the studied biological markers.

### Associations between childhood trauma, plasma biomarkers, and suicide attempt history: a multivariate analysis

To examine how specific biomarkers and childhood trauma are associated with SA history, we conducted independent regression models with CTQ-SF total score and biomarkers as predictors, adjusting for age, sex, BMI, tobacco use, and major psychopharmacological treatment groups. CTQ-SF total score consistently emerged as associated with SA history across all models. Among biological markers, high plasma TSP-2 levels were associated with SA history across all models, with no significant interaction with CTQ-SF total score. Higher levels of MCP-1 were associated with SA history only in baseline models, but this association was not robust to adjustments for psychiatric diagnoses or treatment. Meanwhile, plasma levels of PDGF-BB were not associated with SA history in the initial model, but we observed a significant interaction term with the CTQ-SF score, showing that lower PDGF-BB levels were associated with SA history when self-reported childhood trauma severity was higher. Results from multivariate analyses are presented in Table 2.

**Table 2.** Childhood trauma, plasma biological biomarkers, and lifetime suicide attempts: multivariate analyses

	Model 1		Model 2			Model 3			Model 4		
	Bio	CTQ-SF	Bio	CTQ-SF	Int.	Bio	CTQ-SF	Int.	Bio	CTQ-SF	Int.
Serotonin	0.839 [0.64–1.09] (0.185)	1.027 [1.01–1.04] (<0.001*)	0.618 [0.26–1.44] (0.262)	1.028 [1.01–1.04] (<0.001*)	1.006 [0.99–1.02] (0.452)	0.53 [0.22–1.3] (0.163)	1.022 [1.01–1.04] (0.008*)	1.009 [0.99–1.03] (0.259)	0.644 [0.27–1.52] (0.316)	1.029 [1.01–1.05] (<0.001*)	1.005 [0.99–1.02] (0.535)
MCP–1	1.285 [1.01–1.65] (**0.047**)	1.026 [1.01–1.04] (<0.001*)	1.184 [0.52–2.75] (0.689)	1.026 [1.01–1.04] (<0.001*)	1.002 [0.99–1.02] (0.84)	1.095 [0.46–2.64] (0.838)	1.02 [1–1.04] (0.012*)	1.003 [0.99–1.02] (0.728	1.071 [0.46–2.55] (0.876)	1.026 [1.01–1.04] (0.001*)	1.004 [0.99–1.02] (0.652)
PDGF-AB	0.994 [0.77–1.28] (0.959)	1.025 [1.01–1.04] (0.001*)	0.574 [0.27–1.2] (0.142)	1.026 [1.01–1.04] (0.001*)	1.011 [1–1.03] (0.124)	0.553 [0.25–1.18] (0.13)	1.019 [1–1.04] (0.018*)	1.011 [1–1.03] (0.136)	0.588 [0.27–1.25] (0.17)	1.026 [1.01–1.04] (0.001*)	1.011 [1–1.03] (0.145)
PDGF-BB	1.042 [0.81–1.34] (0.745)	1.027 [1.01–1.04] (<0.001*)	0.452 [0.2–0.98] (**0.046**)	1.03 [1.01–1.05] (<0.001*)	1.016 [1–1.03] (**0.028**)	0.35 [0.15–0.79] (**0.012**)	1.025 [1.01–1.04] (0.005*)	1.02 [1.01–1.04] (0.008*)	0.362 [0.16–0.82] (**0.016**)	1.032 [1.02–1.05] (<0.001*)	1.02 [1.01–1.04] (0.009*)
TSP–1	0.917 [0.72–1.17] (0.491)	1.027 [1.01–1.04] (<0.001*)	0.755 [0.35–1.6] (0.464)	1.027 [1.01–1.04] (<0.001*)	1.004 [0.99–1.02] (0.592)	0.691 [0.31–1.5] (0.351)	1.021 [1.01–1.04] (0.01*)	1.006 [0.99–1.02] (0.467)	0.749 [0.34–1.62] (0.462)	1.027 [1.01–1.04] (<0.001*)	1.004 [0.99–1.02] (0.635)
TSP–2	1.454 [1.12–1.9] (**0.005**)	1.027 [1.01–1.04] (<0.001*)	2.84 [1.26–6.66] (**0.013**)	1.028 [1.01–1.04] (<0.001*)	0.987 [0.97–1] (0.091)	2.725 [1.12–6.75] (**0.028**)	1.022 [1.01–1.04] (0.009*)	0.988 [0.97–1.01] (0.168)	2.754 [1.2–6.58] (**0.019**)	1.028 [1.01–1.04] (<0.001*)	0.988 [0.97–1] (0.127)
CRP	1.248 [0.94–1.67] (0.132)	1.027 [1.01–1.04] (<0.001*)	1.499 [0.69–3.27] (0.308)	1.026 [1.01–1.04] (<0.001*)	0.996 [0.98–1.01] (0.618)	1.477 [0.65–3.35] (0.351)	1.02 [1–1.04] (0.013*)	0.996 [0.98–1.01] (0.617)	1.46 [0.65–3.24] (0.351)	1.027 [1.01–1.04] (<0.001*)	0.996 [0.98–1.01] (0.637)

The table indicates each model and variable of interest its odds ratio, 95% confidence interval (in brackets), and *p*-value (in parentheses). The only effects presented here are the biomarker (Bio), the Childhood Trauma Questionnaire-Short Form score (CTQ-SF), and the interaction between the two (Int.). The *p*-values values are uncorrected, however, *p*-values ≤0.05 after the Benjamini–Hochberg False Discovery Rate (FDR) correction are indicated by an asterisk. The models are as follows: (* denotes an interaction between two terms):

Model 1: SA history ~ biomarker + CTQ-SF + age + gender + Body Mass Index (BMI). Model 2: SA history ~ biomarker*CTQ-SF + age + gender + BMI. Model 3: SA history ~ biomarker*CTQ-SF + age + gender + BMI + Beck Depression Inventory (BDI) score + history of bipolar disorder + history of anxiety disorder + history of eating disorder + history of alcohol/drug abuse. Model 4: SA history ~ biomarker*CTQ-SF + age + gender + BMI + tobacco usage + anxiolytics + antidepressants + antiepileptics + antipsychotics + lithium.

Abbreviations: MCP-1, monocyte chemoattractant protein-1; PDGF-AB, platelet-derived growth factor-AB; PDGF-BB, platelet-derived growth factor-BB; TSP-1, thrombospondin-1; TSP-2, thrombospondin-2; CRP, C-reactive protein.

Bolded values represent to *p*-values <0.05, two-sided.

To further explore the interaction between childhood trauma and PDGF-BB levels on SA history, we employed binomial models adjusted for age, sex, BMI, and tobacco use, assessing associations between CTQ-SF score or PDGF-BB levels with SA history separately, in an additive model, and in an interaction model. Childhood trauma remained strongly associated with SA history across models (*p* < 0.001), while PDGF-BB alone was not associated with SA history in separate or additive models. However, in the interaction model, lower PDGF-BB levels were associated with a higher likelihood of having a SA history (OR = 0.31, 95% CI 0.15–0.64, *p* = 0.002), with significant interaction between childhood trauma severity and PDGF-BB levels (OR = 1.02, 95% CI 1.01–1.04, *p* = 0.001) (see Table 3).

**Table 3.** Childhood trauma, PDGF-BB, and lifetime suicide attempt history: a multivariate analysis

Characteristic	Model 1, *N* = 311			Model 2, *N* = 311			Model 3, *N* = 311			Model 4, *N* = 311		
	OR	95% CI	*p*-Value	OR	95% CI	*p*-Value	OR	95% CI	*p*-Value	OR	95% CI	*p*-Value
CTQ-SF score	1.04	1.02, 1.05	<0.001*	–	–	–	1.04	1.02, 1.05	<0.001*	1.04	1.03, 1.06	<0.001*
Sex												
Female	–	–	–	–	–	–	–	–	–	–	–	–
Male	0.91	0.53, 1.57	0.745	0.78	0.46, 1.30	0.338	0.93	0.54, 1.60	0.787	0.95	0.54, 1.66	0.848
Age	0.98	0.96, 1.00	0.095	0.99	0.97, 1.01	0.156	0.98	0.96, 1.00	0.089	0.98	0.96, 1.00	0.041
BMI	1.04	0.99, 1.10	0.15	1.04	0.99, 1.09	0.115	1.04	0.99, 1.09	0.153	1.04	0.98, 1.09	0.188
Tobacco												
No	–	–	–	–	–	–	–	–	–	–	–	–
Yes	1.57	0.97, 2.57	0.069	1.73	1.08, 2.77	0.022	1.59	0.97, 2.60	0.065	1.56	0.95, 2.58	0.08
PDGF-BB	–	–	–	0.89	0.71, 1.12	0.305	0.95	0.74, 1.21	0.679	0.31	0.15, 0.64	0.002*
CTQ-SF score * PDGF-BB	–	–	–	–	–	–	–	–	–	1.02	1.01, 1.04	0.001*

Abbreviations: CI, confidence interval; OR, odds ratio.

The models are as follows: (* denotes an interaction between two terms):

Model 1: History of SA ~ CTQ-SF + age + sex + BMI + tobacco usage.

Model 2: History of SA ~ PDGF-BB + age + sex + BMI + tobacco usage.

Model 3: History of SA ~ CTQ-SF + PDGF-BB + age + sex + BMI + tobacco usage.

Model 4: History of SA ~ CTQ-SF * PDGF-BB + age + sex + BMI + tobacco usage.

Abbreviations: CTQ-SF, Childhood Trauma Questionnaire-Short Form; PDGF-BB, platelet-derived growth factor-BB; BMI, body mass index.

Bolded values represent to *p*-values <0.05, two-sided.

**Figure 1.**
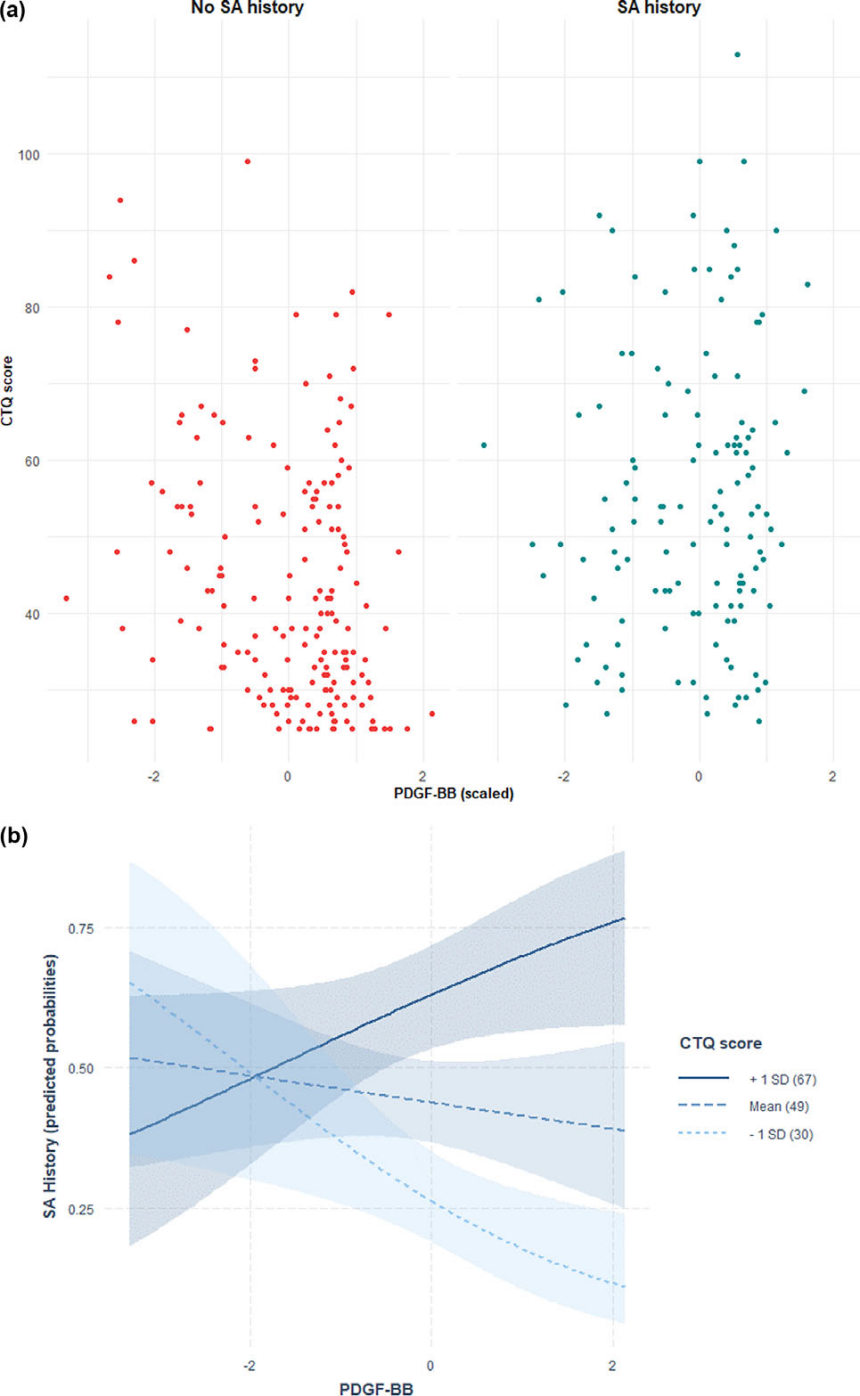
(A) Distribution of CTQ-SF scores in relation to PDGF-BB levels and suicide attempt (SA) history. Scatter plot showing Childhood Trauma Questionnaire-Short Form (CTQ-SF) scores in relation to plasma PDGF-BB levels (z-scored) in individuals with (right, blue) and without (left, red) a history of suicide attempts. Each point represents a participant. (B) Predicted probabilities of suicide attempt history based on the adjusted interaction model between PDGF-BB and CTQ-SF score. Predicted probabilities of suicide attempt history as a function of plasma PDGF-BB levels, modelled via multivariate logistic regression with an interaction term between PDGF-BB and Childhood Trauma Questionnaire-Short Form (CTQ-SF) scores, adjusted for age, sex, and body mass index. Lines indicate CTQ-SF at +1 SD (solid), mean (dashed), and −1 SD (dotted), with 95% confidence intervals (shaded).

**Figure 2.**
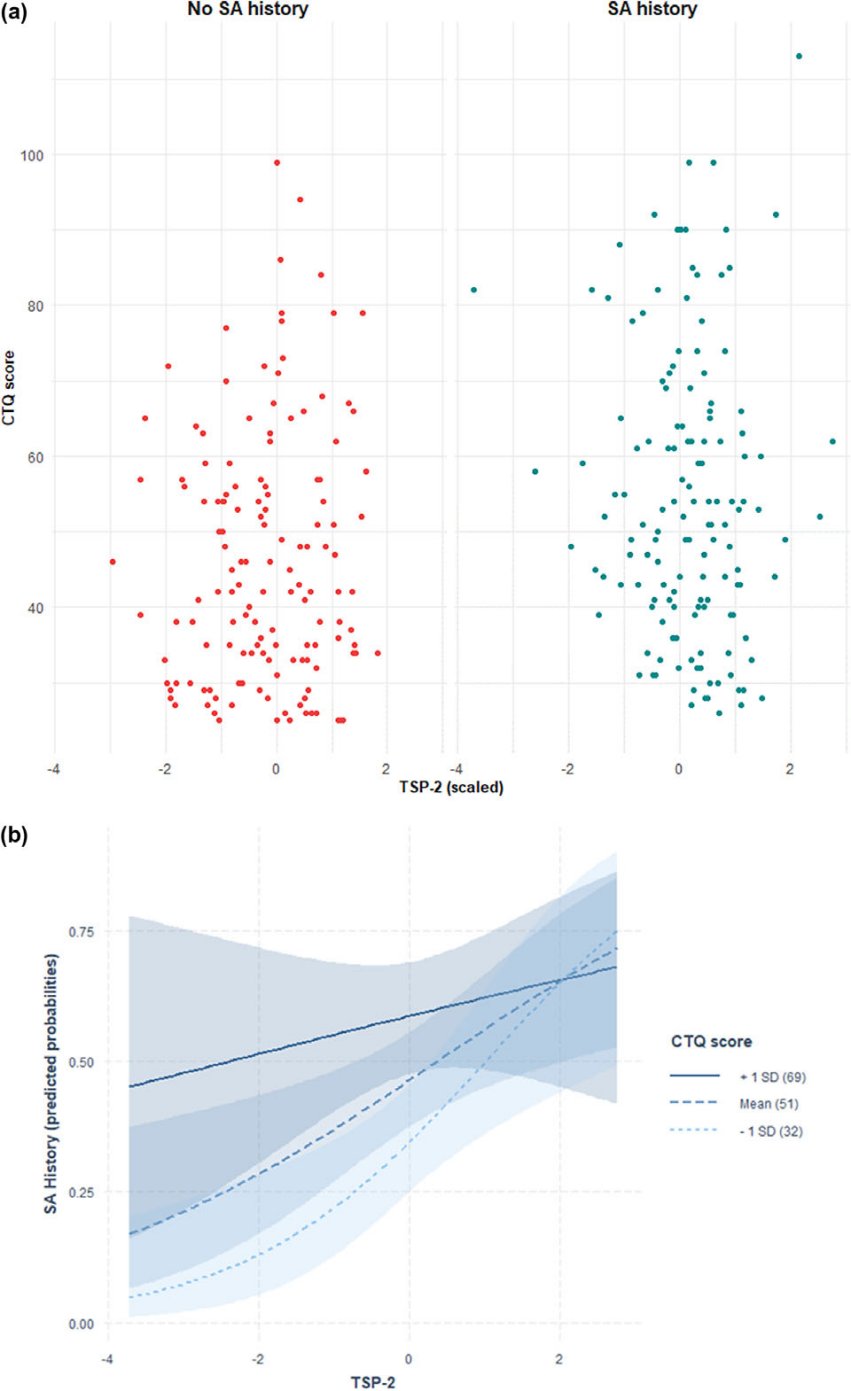
(A) Distribution of CTQ-SF scores in relation to TSP-2 levels and suicide attempt (SA) history. Scatter plot showing Childhood Trauma Questionnaire-Short Form (CTQ-SF) scores in relation to plasma TSP-2 levels (z-scored) in individuals with (right, blue) and without (left, red) suicide attempt (SA) history. Each point represents a participant. (B) Predicted probabilities of suicide attempt history based on the adjusted interaction model between TSP-2 and CTQ-SF score. Predicted probabilities of suicide attempt history based on an interaction model between plasma TSP-2 and Childhood Trauma Questionnaire-Short Form (CTQ-SF) scores, adjusted for age, sex, and body mass index. Lines represent CTQ-SF at +1 SD (solid), mean (dashed), and −1 SD (dotted), with 95% confidence intervals (shaded).

Distribution of CTQ-SF scores in relation to PDGF-BB and TSP-2 levels with SA history are depicted in Figures 1A and 2A, while predicted probabilities of SA history based on the adjusted interaction model between PDGF-BB and TSP-2 with CTQ-SF score are depicted in Figures 1B and 2B.

### Stratified analyses by childhood trauma

As a sensitivity analysis, we looked at whether plasma biomarkers are associated with SA history in people with childhood trauma and those without. Instead of controlling for childhood trauma in the model, stratified analyses were conducted in patients with at least one form of trauma and those without.

Among patients with childhood trauma, none of the biomarkers showed significant associations with SA history across the models (see Table 4).

**Table 4.** Stratified analysis by childhood trauma: patients with at least one childhood trauma type

	Model 1		Model 2		Model 3	
	AIC	Bio	AIC	Bio	AIC	Bio
Serotonin	261.85	0.894 [0.67–1.19] (0.448)	252.01	0.927 [0.68–1.27] (0.636)	267.84	0.857 [0.61–1.2] (0.368)
PDGF-AB	275.40	1.129 [0.84–1.53] (0.429)	266.37	1.104 [0.8–1.52] (0.544)	282.49	1.132 [0.83–1.55] (0.434)
PDGF-BB	262.94	1.168 [0.88–1.55] (0.279)	252.97	1.175 [0.87–1.58] (0.284)	269.09	1.18 [0.88–1.59] (0.266)
TSP–1	278.61	0.961 [0.72–1.29] (0.792)	270.74	0.983 [0.72–1.34] (0.914)	285.05	0.927 [0.68–1.26] (0.624)
TSP–2	275.94	1.282 [0.96–1.74] (0.102)	267.36	1.353 [0.98–1.89] (0.07)	282.19	1.316 [0.97–1.8] (0.081)
CRP	281.05	1.121 [0.8–1.57] (0.502)	271.90	1.102 [0.78–1.57] (0.589)	287.38	1.125 [0.8–1.59] (0.501)

The models are as follows:

Model 1: SA history ~ biomarker + age + gender + body mass index (BMI). Model 2: SA history ~ biomarker + age + gender + BMI + BDI score + history of bipolar disorder + history of anxiety disorder + history of eating disorder + history of alcohol/drug abuse. Model 3: SA history ~ biomarker + age + gender + BMI + tobacco usage + anxiolytics + antidepressants + antiepileptics + antipsychotics + lithium.

Abbreviations: MCP-1, monocyte chemoattractant protein-1; PDGF-AB, platelet-derived growth factor-AB; PDGF-BB, platelet-derived growth factor-BB; TSP-1, thrombospondin-1; TSP-2, thrombospondin-2; CRP, C-reactive protein.

In contrast, for patients without childhood trauma, several findings emerged. Higher PDGF-BB levels had a protective effect in Model 2 (*p* = 0.05), though this effect diminished and lost significance in the most adjusted model, suggesting it may reduce the risk of SA in individuals without childhood trauma, but its impact weakened when psychiatric treatment factors were considered. Plasma TSP-2 levels were associated with SA history across all models, particularly in Model 2 (*p* = 0.012) and Model 3 (*p* = 0.016). Other analysed biomarkers did not display consistent or significant effects across the models (see Table 5).

**Table 5.** Stratified analysis by childhood trauma: patients with no childhood trauma

	Model 1		Model 2		Model 3	
	AIC	Bio	AIC	Bio	AIC	Bio
Serotonin	110.71	0.651 [0.37–1.11] (0.122)	112.34	0.667 [0.37–1.19] (0.173)	115.96	0.614 [0.31–1.15] (0.142)
PDGF-AB	123.64	0.806 [0.52–1.24] (0.323)	125.58	0.776 [0.48–1.24] (0.288)	129.02	0.769 [0.47–1.23] (0.275)
PDGF-BB	111.18	0.701 [0.42–1.15] (0.156)	110.27	0.577 [0.33–0.99] (**0.05**)	115.06	0.609 [0.34–1.05] (0.077)
TSP–1	125.11	0.853 [0.54–1.32] (0.479)	126.31	0.793 [0.48–1.28] (0.348)	130.24	0.826 [0.51–1.32] (0.429)
TSP–2	119.54	1.885 [1.13–3.36] (**0.021**)	119.63	2.19 [1.24–4.3] (**0.012**)	124.24	2.011 [1.17–3.71] (**0.016**)
CRP	126.48	1.443 [0.85–2.5] (0.178)	127.49	1.372 [0.76–2.52] (0.293)	131.32	1.45 [0.82–2.62] (0.205)

The models are as follows:

Model 1: SA history ~ biomarker + age + gender + body mass index (BMI). Model 2: SA history ~ biomarker + age + gender + BMI + BDI score + history of bipolar disorder + history of anxiety disorder + history of eating disorder + history of alcohol/drug abuse. Model 3: SA history ~ biomarker + age + gender + BMI + tobacco usage + anxiolytics + antidepressants + antiepileptics + antipsychotics + lithium.

Abbreviations: MCP-1, monocyte chemoattractant protein-1; PDGF-AB, platelet-derived growth factor-AB; PDGF-BB, platelet-derived growth factor-BB; TSP-1, thrombospondin-1; TSP-2, thrombospondin-2; CRP, C-reactive protein.

Bolded values represent to *p*-values <0.05, two-sided.

## Discussion

This study aimed to investigate the association between childhood trauma, immune-inflammatory and vascular homeostasis-related biological markers, and SA history in adults with mood disorders. Consistent with previous literature [4, 5], we found a robust association between childhood trauma and SA history. Moreover, this study provides novel insights into the potential biological pathways potentially involved in SA. Notably, we observed a positive association between plasma TSP-2 levels and SA history, independent of childhood trauma history, and an interaction between PDGF-BB and childhood trauma severity in their association with SA history. These findings reinforce the potential role of these markers in the pathogenesis of suicidal behaviours, and suggest that PDGF-BB may modulate the impact of childhood trauma on the risk of SA.

The association between elevated MCP-1 levels and SA history was significant in unadjusted models but weakened and lost its significance after adjusting for psychiatric diagnoses and treatment, suggesting that this relationship may be mediated by clinical factors. This aligns with a previous systematic review that reported the associations of multiple chemokines, including MCP-1, with psychiatric disorders [25]. Similarly, Tolf et al. [26] reported significantly higher serum MCP-1 levels in psychiatric patients compared to healthy controls. In contrast, the absence of significant associations between CRP levels and lifetime history of SA diverges from findings in some previous studies, which have reported associations between higher CRP levels and SA history [14, 20, 27]. In our study, CRP levels were numerically higher in the suicide attempters group. However, the absence of a statistically significant association may reflect limited statistical power, given the relatively small effect sizes reported in earlier research. Moreover, prior studies have suggested that CRP is more strongly associated with recent SA rather than distal SA history, which our study did not differentiate, as we do not have data on the date of the SA [20, 27]. Notably, elevated CRP levels are primarily linked to acute or subacute systemic inflammation, emphasizing that this marker predominantly reflects recent inflammatory activity. As such, current CRP levels may have limited utility as retrospective indicators of distal SA history.

Beyond these results, our findings provide compelling evidence for the association of TSP-2 and PDGF-BB with SA history, emphasizing their potential as biomarkers for suicide risk. This study builds on our previous study, which reported an association between higher plasma levels of TSP-2 and recent SA history in a sample of 266 patients with mood disorders [20]. By expanding the sample size, we demonstrated that higher TSP-2 levels were consistently associated with a lifetime history of SA. Notably, this association remained significant even among individuals without a history of childhood trauma, suggesting the involvement of an underlying biological mechanism linking TSP-2 to suicidal behaviour that is independent of adverse early-life experiences.

Despite the limited research on TSP-2 in psychiatry, its involvement in platelet activity, vascular function, and neuroinflammation [28] supports its potential role as a marker of interest. Elevated TSP-2 levels have also been associated with physical and psychological pain conditions, both of which are key factors contributing to suicide risk [29, 30]. Beyond its psychiatric implications, TSP-2 has been implicated in a range of medical conditions, including liver disease, cardiovascular disorders, and various cancers [31–33]. Its immunomodulatory properties are well-documented, including promoting dendritic cell maturation, cytokine production, and T-cell differentiation in parasitic diseases [34], as well as exerting anti-inflammatory effects in allergic asthma by reducing airway hyperactivity and lung inflammation [35]. By demonstrating an association between TSP-2 and SA history, our findings extend the relevance of this biomarker to the field of psychiatry, and implicating platelet function, vascular homeostasis, and neuroinflammation in the biological underpinnings of suicidal behaviours.

In parallel, our results highlight the role of PDGF-BB as a potential protective factor against SA, particularly in the context of severe childhood trauma. While PDGF-BB alone was not significantly associated with a lifetime history of SA, its interaction with childhood trauma severity revealed a buffering effect that mitigated the association between childhood trauma and SA history. These findings are consistent with emerging evidence supporting the role of PDGF-BB in promoting resilience and adaptive responses to stress. For instance, experimental models have shown that endothelial expression of PDGF-BB promotes pericyte migration and stabilization in the brain, supporting resilient and adaptive behaviours [36]. Similarly, PDGF-BB has been shown to regulate neurogenesis and reverse depressive-like behaviours in hippocampal circuitry [37]. These neurovascular and neuroplastic effects suggest that PDGF-BB may mediate biological responses that counteract the detrimental effects of early-life adversity. Beyond its neurobiological functions, PDGF-BB’s role in cellular adaptive processes further underscores its relevance. Pestana et al. [38] demonstrated that PDGF-BB acts in an autocrine or paracrine manner to stimulate cellular proliferation and plays a critical role in nicotine-induced mitogenic actions on human aortic smooth muscle cells. Taken together, these findings position PDGF-BB as a mediator of resilience that might mitigate the long-term impact of early-life adversity on suicidal behaviour risk.

Overall, these findings highlight the unique roles of TSP-2 and PDGF-BB in suicidal behaviour in the context of childhood trauma. Increased TSP-2 might reflect underlying biological processes associated with SA vulnerability, independent of childhood trauma, whereas PDGF-BB might have a protective effect by interacting with childhood trauma severity to buffer its impact on suicidal behaviour. Notably, previous studies indicate that individuals with childhood trauma not only have an increased risk of suicidal behaviours, but may also exhibit a “male depression” phenotype characterized by lowered stress tolerance, irritability, impulsive or aggressive behaviour, and substance-related behaviours [39]. Although we did not specifically measure these potentially intermediate phenotypes, it is possible that PDGF-BB reflects a neurobiological predisposition to such features, which may ultimately contribute to SA risk. Future studies are needed to confirm these mechanisms and clarify whether targeting these intermediate phenotypes could inform more effective prevention and intervention strategies.

### Limitations and strengths

The study has several important limitations. Firstly, data were collected exclusively at a single clinical centre, requiring validation in an independent cohort. Second, it is a cross-sectional study and does not allow establishing causal relationships. Ideally, future studies should explore the longitudinal relationships between childhood trauma, PDGF, and TSP, their changes, and the risk of suicidal behaviours to better understand causality and temporal dynamics. There is a need for predictive biomarkers and robust models, ideally integrating biological factors, in advancing suicide risk assessment and prevention strategies [40, 41]. Additionally, the reliance on self-reported measures of childhood trauma may be subject to recall bias. Furthermore, the sample was limited to adults with depression, which restricts the generalizability of the findings to other populations, such as adolescents or adults without depression. Expanding research to more diverse populations would provide a broader understanding of these relationships. Finally, while this study focused on SA, it did not assess differences among subtypes of SA. Given the reported differences in biological underpinnings and role of childhood trauma in early-onset, high-lethality SA [9], future large-scale studies would benefit from more granular analyses differentiating SA subtypes, including lethality, violence, impulsivity, and age of onset.

Despite these limitations, the study includes several strengths. Primarily, it innovatively focuses on novel biomarkers (e.g., PDGF-BB, TSP-2) that have not been extensively investigated in the context of suicidal behaviour. This novel approach provides valuable insights into the potential biological mechanisms underlying the link between early life adversity and suicide risk. By integrating established risk factors (childhood trauma, traditional inflammatory markers) with emerging biomarkers (platelet-derived growth factors and thrombospondins), the study bridges existing knowledge gaps and expands our understanding of suicidal behaviour. These novel insights into the role of specific biomarkers offer promising avenues for future research and potential clinical applications. Furthermore, the study adjusts for a wide range of covariates, including psychiatric diagnoses, depression severity, psychopharmacological treatments, and lifestyle factors (e.g., tobacco use), ensuring that the observed associations are not confounded by these variables.

## Conclusion

Our findings emphasize the critical role of childhood trauma in the risk of SA and identify TSP-2 and PDGF-BB as promising biomarkers, particularly within trauma-stratified groups. Higher plasma levels of TSP-2 were associated with a history of SA, independent of childhood trauma severity. In contrast, while PDGF-BB alone was not associated with SA history, its interaction with severe childhood trauma revealed a protective effect, suggesting that PDGF-BB may buffer the adverse impact of early-life adversity on SA. These results enhance our understanding of the complex interplay between early-life adversity, vascular homeostasis-related biological markers, and suicidal behaviour. Clinically, incorporating TSP-2 and PDGF-BB measures alongside childhood trauma history could improve risk stratification and guide more tailored suicide prevention strategies. Furthermore, understanding how PDGF-BB interacts with childhood trauma may inform novel therapeutic approaches aimed at mitigating the long-term consequences of early-life adversity. By identifying potential mechanisms underlying suicide risk, this study lays the groundwork for the development of biomarker-informed, personalized strategies for suicide prevention. Future research should focus on validating these biomarkers in longitudinal studies and exploring their potential in guiding targeted interventions, ultimately advancing personalized, biomarker-informed suicide prevention strategies.

## Supporting information

10.1192/j.eurpsy.2025.10029.sm001 García-Fernández et al. supplementary material García-Fernández et al. supplementary material

## Supplementary material

The supplementary material for this article can be found at http://doi.org/10.1192/j.eurpsy.2025.10029.
