# Supplementary material for: Independent and interactive associations of inflammation, vascular homeostasis markers, and childhood trauma with suicide attempt history
Source: Eur Psychiatry. 2025 May 27;68(1):e71. doi: 10.1192/j.eurpsy.2025.10029 (PMC12260722; doi:10.1192/j.eurpsy.2025.10029)
Supplement: García-Fernández et al. supplementary material [file S0924933825100291sup001.docx]

| **Biomarker** | **Adj. Corr (CTQ-SF)** | **p-value (CTQ-SF)** | **Adj. Corr (BDI)** | **P-value (BDI)** |
| --- | --- | --- | --- | --- |
| **Serotonin** | -0.077 (-0.186 - 0.034) | 0.173 | -0.225 (-0.331 - -0.114) | <0.001 |
| **Platelet-Derived Growth Factor (PDGF-AB)** | -0.006 (-0.117 - 0.104) | 0.91 | -0.077 (-0.178 - 0.027) | 0.147 |
| **Platelet-Derived Growth Factor (PDGF-BB)** | -0.106 (-0.219 - 0.009) | 0.07 | -0.109 (-0.217 – 0) | 0.05 |
| **Thrombospondin 1 (TSP-1)** | 0.004 (-0.106 - 0.114) | 0.941 | -0.065 (-0.165 - 0.036) | 0.209 |
| **Thrombospondin 2 (TSP-2)** | -0.027 (-0.135 - 0.082) | 0.625 | -0.030 (-0.138 - 0.078) | 0.583 |
| **C-reactive protein (CRP)** | -0.050 (-0.151 - 0.053) | 0.345 | 0.013 (-0.092 - 0.118) | 0.806 |

**Supplementary Table 1.** Partial Spearman Correlation between biomarkers, childhood trauma and depression.

Abbreviations: BDI: Beck Depression Inventory; CTQ-SF: Childhood Trauma Questionnaire-Short Form.
